# Simulation-Driven Machine Learning for Predicting Stent Expansion in Calcified Coronary Artery

**DOI:** 10.3390/app10175820

**Published:** 2020-08-22

**Authors:** Pengfei Dong, Guochang Ye, Mehmet Kaya, Linxia Gu

**Affiliations:** Department of Biomedical and Chemical Engineering, Florida Institute of Technology, Melbourne 32901, Australia

**Keywords:** calcified coronary artery, machine learning, support vector regression (SVR), stent expansion, finite element (FE) method

## Abstract

In this work, we integrated finite element (FE) method and machine learning (ML) method to predict the stent expansion in a calcified coronary artery. The stenting procedure was captured in a patient-specific artery model, reconstructed based on optical coherence tomography images. Following FE simulation, eight geometrical features in each of 120 cross sections in the pre-stenting artery model, as well as the corresponding post-stenting lumen area, were extracted for training and testing the ML models. A linear regression model and a support vector regression (SVR) model with three different kernels (linear, polynomial, and radial basis function kernels) were adopted in this work. Two subgroups of the eight features, i.e., stretch features and calcification features, were further assessed for the prediction capacity. The influence of the neighboring cross sections on the prediction accuracy was also investigated by averaging each feature over eight neighboring cross sections. Results showed that the SVR models provided better predictions than the linear regression model in terms of bias. In addition, the inclusion of stretch features based on mechanistic understanding could provide a better prediction, compared with the calcification features only. However, there were no statistically significant differences between neighboring cross sections and individual ones in terms of the prediction bias and range of error. The simulation-driven machine learning framework in this work could enhance the mechanistic understanding of stenting in calcified coronary artery lesions, and also pave the way toward precise prediction of stent expansion.

## 1. Introduction

Heavily calcified arteries are commonly associated with suboptimal stenting outcomes, including stent underexpansion and malapposition [1,2], which have been associated with chronic complications, such as restenosis and thrombosis [1,3–6]. The calcification characteristics (such as calcification angle, area, calcium scores, etc.) are associated with stenting outcomes in a clinical setting [1,7,8]. Calcium score, assessed by intravascular ultrasound (IVUS) or optical coherence tomography (OCT), has been commonly used as a biomarker for clinical classification and risk management [9–11]. A retrospective study showed that lesions with a higher calcium score often require post-dilation using a larger non-compliant balloon at a higher inflation pressure [7]. Another retrospective study has shown that stenting expansion is related to the calcification angle and area assessed with OCT images [1]. An OCT-based calcium scoring system was proposed to predict the possibility of stent underexpansion [8]. The score depends on the maximum angle, thickness, and the length of the calcification, with critical values of 180°, 0.5 mm, and 5 mm, respectively. These retrospective studies provide a qualitative relationship between calcification characteristics and stent expansion.

Mechanical tests and in vitro/silico studies have also been performed to quantitatively inspect the mechanism of the stent–artery interaction in a calcified artery. Nanoindentation tests of calcified arteries showed that the Young’s modulus of calcification is 690 MPa, which is much larger than that of fibrotic tissue, which is in the range of 50 to 980 kPa [12,13]. Uniaxial tensile tests of calcified artery segments along the circumferential direction showed that a larger calcification volume reduces the stretch capability of the artery [14,15]. Our previous work has demonstrated that stenting induces larger stretch in the fibrotic tissue than that in calcification, which contributes to lumen gain [2,16]. Specifically, the calcification angle determines the arterial stretch and thus lumen gain.

Machine learning (ML) methods have been integrated with engineering approaches for mechanistic prediction. Combined with finite element (FE) or molecular dynamic simulation, ML methods have been used to predict the Young’s modulus of composite materials [17,18]. Simulation could provide enriched datasets in the computational domain, such as the stress/strain inside the artery, which is difficult to measure using experimental techniques. These datasets could enhance the training of the ML, which would then help to optimize virtual tests. On the other hand, ML has been used for image processing, pattern identification [19,20], and predicting in-stent restenosis based on acute stenting outcomes (lumen area, shape, etc.), as well as demographic characteristics such as diabetes and other related diseases [21,22]. However, ML has not been integrated with FE methods yet to predict stent expansion.

In this work, we developed simulation-driven ML models to predict the post-stenting lumen area in a calcified coronary artery. An in-silico stenting procedure was performed in a patient-specific coronary artery model reconstructed from OCT images in our previous work [16]. The mechanistic understanding from FE simulation inspired us to select stretch features besides classical calcification features. Eight pre-stenting features and the post-stenting lumen area were used to train and test ML models. The accuracy of the ML models was evaluated in terms of the prediction error, i.e., the percentage difference between the predicted lumen area and the one obtained from FE simulation. Two subgroups, stretch features and calcification features, were compared to study the influence of feature selection on the prediction. In addition, the influence of neighboring cross sections on the prediction was also investigated by averaging each feature over eight neighboring cross sections.

## 2. Materials and Methods

**Figure 1.**
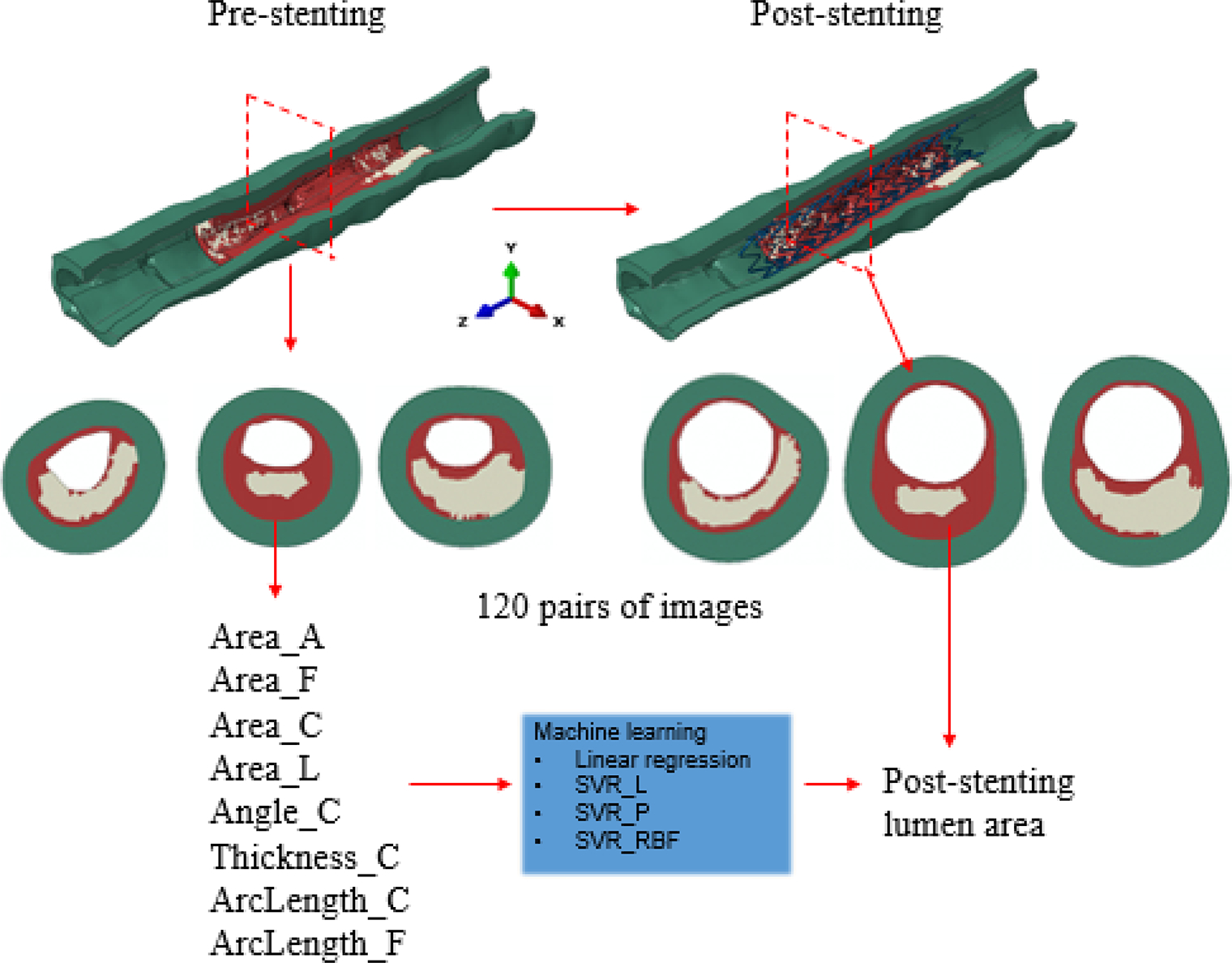
Workflow of simulation-driven machine learning (ML) methods to predict post-stenting lumen area in a calcified coronary artery.

The workflow integrating the FE method and the ML method is depicted in Figure 1. A patient-specific coronary artery model was reconstructed from OCT images and used for simulating the stent deployment, as described in our previous work [16]. Briefly, the calcified artery model was reconstructed based on 131 OCT images with a pixel size of 12.5 μm and spacing of 200 μm. Both calcification and fibrotic tissue were considered as components of plaque, corresponding to the late stage of a calcified artery. Stent deployment was implemented in an OCT-based patient-specific coronary artery model through the FE model as described in the next paragraph. There were 120 cross sections with spacing of 0.1 mm extracted from the FE model, within the stented region (i.e., 7 mm away from both ends of the artery) at pre- and post-stenting stages, respectively. From pre-stenting images, eight features were extracted: area of the artery (Area_A), fibrotic tissue (Area_F), calcification (Area_C) and lumen (Area_L), calcification angle (Angle_C) and maximum thickness of the calcification (Thickness_C), inner circumferential length of the calcification and fibrotic tissue (ArcLength_C and ArcLength_F, respectively). All these features were measured using an in-house code developed in Python 3. Correlational studies were performed to investigate the dependent relationships between different features. The centroid of the lumen was identified for measuring the calcification angle. From post-stenting images, the lumen area was extracted at each cross section, which was also the output parameter from ML models. In total, 120 pairs of datasets, eight pre-stenting features as the input variables, and one lumen area value as the output variable, were obtained for the regression learning and prediction. Specifically, the first 90 pairs of datasets were selected for training, and the rest of the 30 pairs were used for testing. A support vector regression (SVR) model with three different kernels, linear, polynomial, and radial basic function (RBF) (SVR_L, SVR_P, and SVR_RBF, respectively), were adopted and compared with a linear regression (LR) model.

The artery had a length of 26 mm, which was 5 mm longer than the stent at each end. An Express stent with a length of 16 mm was deployed in the artery model. The hyperelastic material behavior was adopted to describe the mechanical behavior of the tissue. A reduced third-order polynomial was defined for the strain density function of the material:

(1) $$U=\sum_{i,j=1}^{3}C_{ij}{\left(I_{1}-3\right)}^{i}{\left(I_{2}-3\right)}^{j}$$

(2) $$I_{1}=\lambda_{1}^{2}+\lambda_{2}^{2}+\lambda_{3}^{2}$$

(3) $$I_{2}=1/\lambda_{1}^{2}+1/\lambda_{2}^{2}+1/\lambda_{3}^{2}$$

where *I*_*1*_ and *I*_*2*_ are the first and second invariants of the Cauchy–Green deformation tensor, and λ_1_, λ_2_, and λ_3_ are the principal stretches. The coefficients *C*_*ij*_ are adopted in the literature [23,24], as summarized in Table 1. In addition, a perfect plastic behavior was adopted for the fibrotic plaque with the yield stress of 0.07 MPa and the corresponding yield strain of 34% [25]. The stent material was 316L stainless steel, with a Young’s modulus of 190 GPa, Poisson’s ratio of 0.3, and yield strength of 207 MPa [24]. Symmetric constraints were enforced at both ends of the artery. The stent was first crimped from its nominal outer diameter of 3 mm into 1 mm to mimic its delivery state. At the stenotic location, the stent was radially expanded to a diameter of 3.3 mm, which compressed and pushed the stenotic lesion outwards [26]. After unloading, the stent recoiled to its final shape. A frictionless contact was enforced between the stent and the lesion [27]. The model was solved using the commercial software Abaqus (Dassault Systèmes Simulia Corp., Providence, RI, USA). The FE simulation was validated against the OCT measurements of lumen area, lumen shape, and malapposition of stent struts.

Linear regression is one of the fundamental supervised machine learning algorithms. For a given dataset (y_i_, x_i1_, x_i2_, . . . x_i8_), i represents 90 sets of training data, x_i1_ to x_i8_ represent eight features, and y_i_ is the stent-induced lumen area. The linear model is:

(4) y=Xw+ε

where *w* is the regression coefficient and ε is the correction term. Both are obtained using the least squares method to minimize the error between the predicted value and the measured value:

(5) $$\min\sum_{i=0}^{n}{\left(y_{i}-x_{i}w\right)}^{2}$$

**Figure 2.**
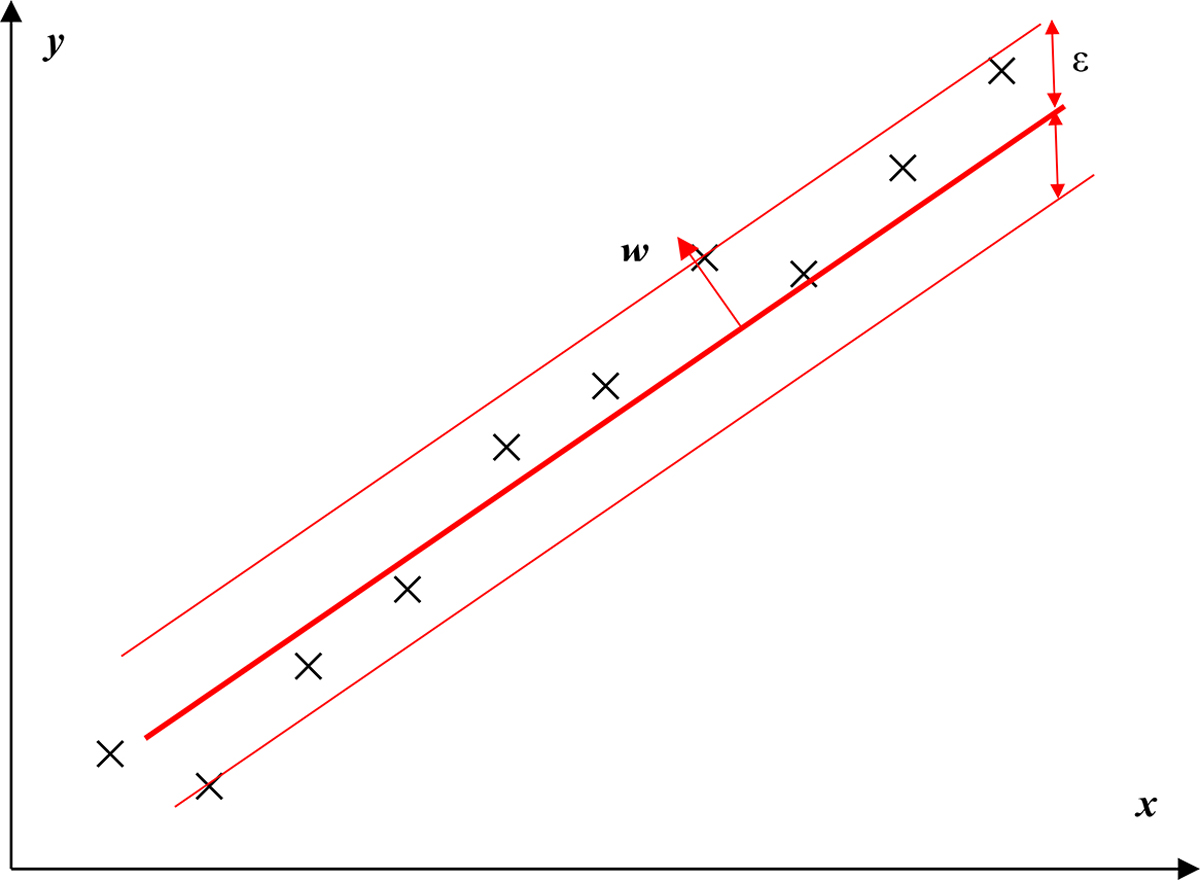
Schematic of support vector regression method.

On the other hand, the SVR method regulates the error to a certain degree as illustrated in Figure 2. The objective function of the SVR method is to minimize the margin of the hyperplane, especially the l2-norm of the normal vector *w*:

(6) $$\min\frac{1}{2}\|w\|$$

under the constraints:

(7) $$\{\begin{array}{l} y_{i}-wx_{i}-b\leq \varepsilon \\ wx_{i}+b-y_{i}\geq \varepsilon \end{array}$$

Besides the linear kernel, the SVR models could transfer the feature space into a higher dimensional feature space with nonlinear kernel functions, such as polynomial and RBF kernels. All machine learning models were developed within Python 3.

The accuracy of these four ML models was evaluated in terms of the percentage error between the predicted value and the measured value from the FE model. The influence of the feature selection and neighboring cross sections on prediction accuracy were further studied using SVR_P. Two subgroups, stretch features (Area_L, ArcLength_C, and ArcLength_F) and calcification features (Area_C, Angle_C, and Thickness_C), were selected for inspecting the influence of feature selection on the prediction accuracy. In addition, the influence of neighboring cross sections on the prediction accuracy was also investigated by using the average value of each feature over eight neighboring cross sections (i.e., four cross sections, if any, immediately before and after the current cross section) for training and testing the regression model.

Statistical analysis was performed with *p*-value < 0.05 considered statistically significant. The ANOVA test was used to compare the means among more than two groups of the prediction errors, and a simple t-test was used to compare the means between two groups.

## 3. Results

**Figure 3.**
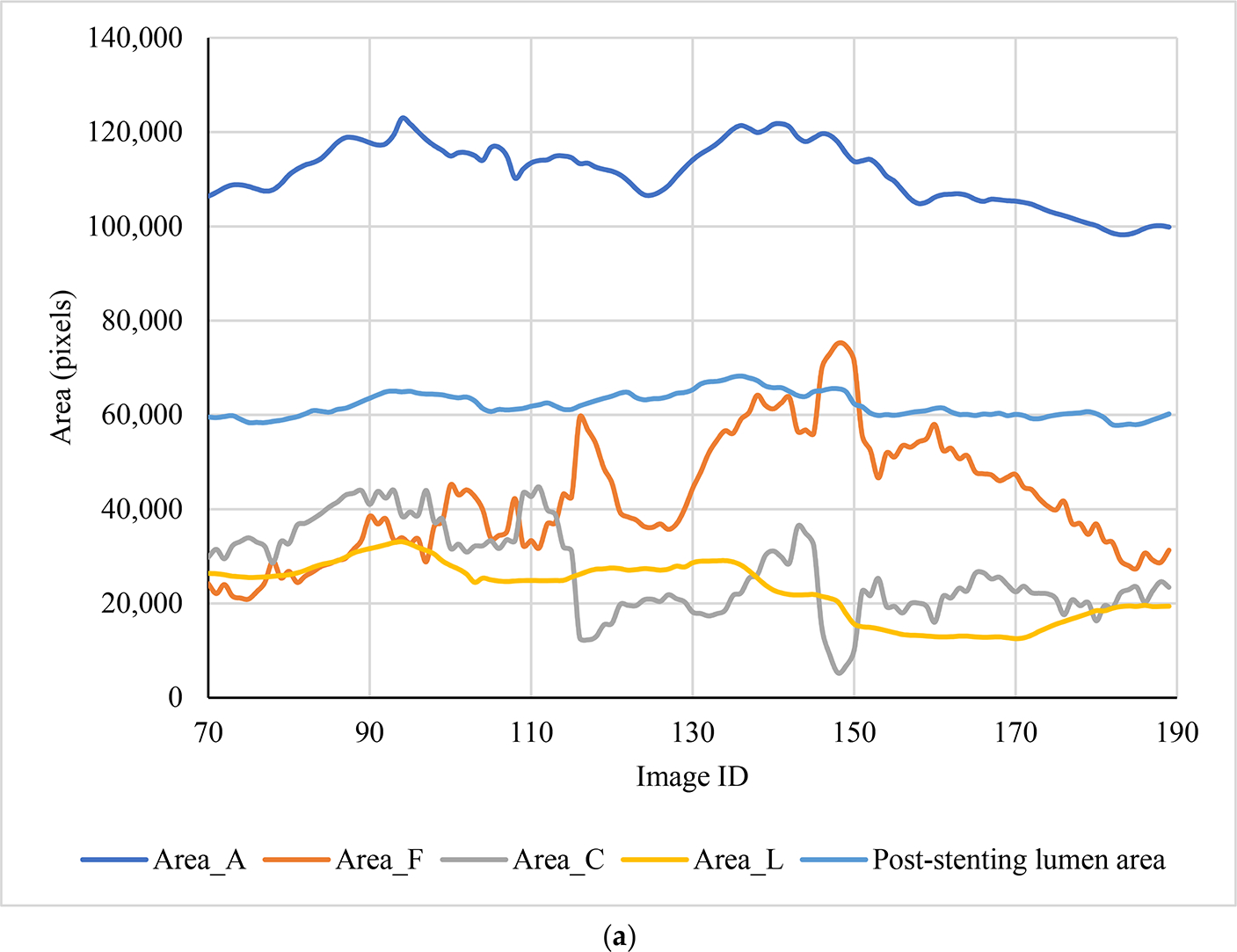

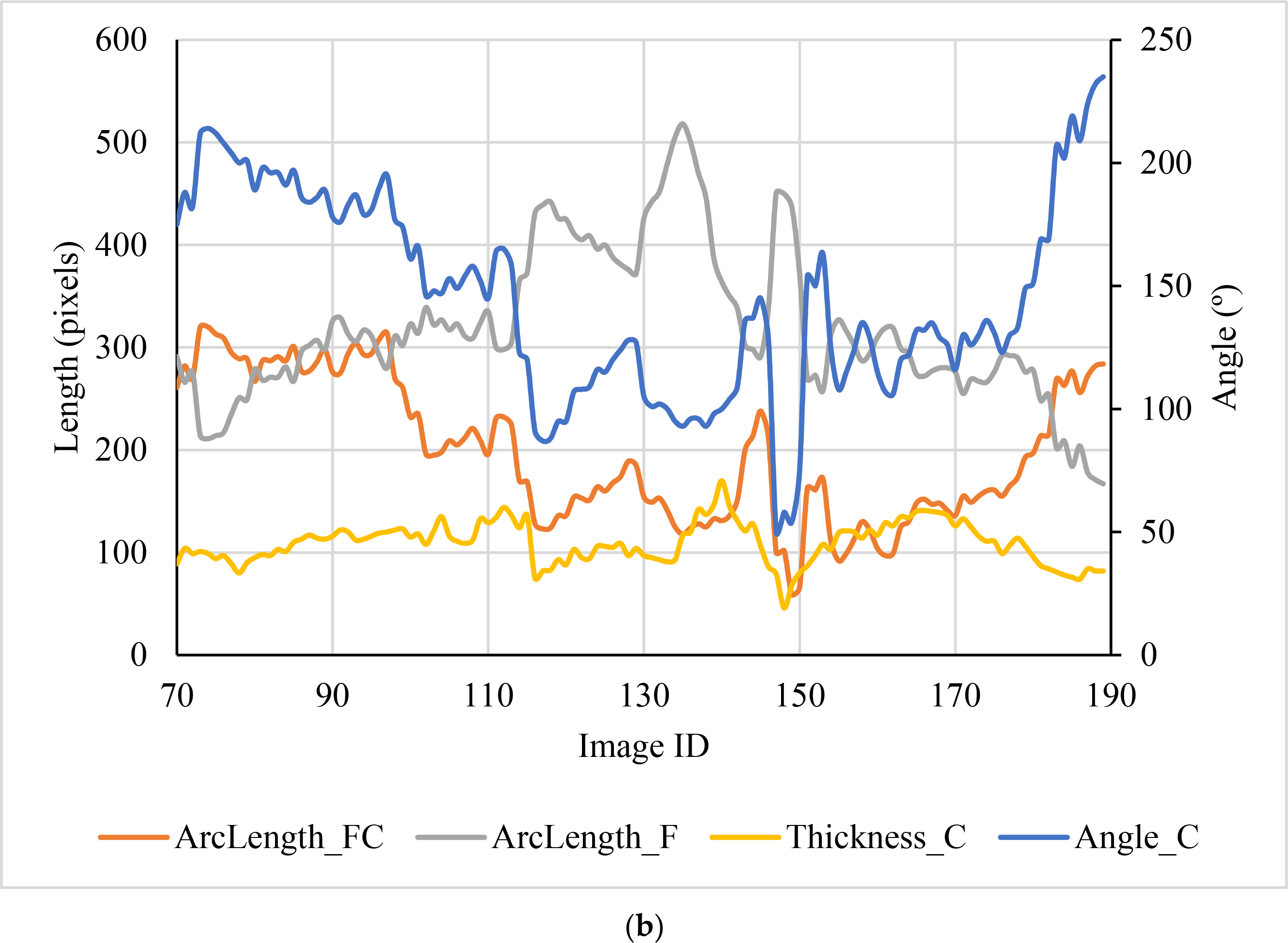
Eight features at 120 cross sections as well as the corresponding post-stenting lumen area: (**a**) area features, and (**b**) length features and calcification angle.

A total of 120 pairs of pre- and post-stenting cross sections were extracted from the FE models for training and testing regression models. Four area features (Area_A, Area_F, Area_C, Area_L, and stenting expansion), along with the calculated post-stenting lumen area, are depicted in Figure 3a. The length features (ArcLength_C, ArcLength_F, and Thickness_C) and the calcification angle are shown in Figure 3b. It is clear that the major features of these cross sections are different due to the presence of heterogeneous calcifications. The calcification area is associated with its angle (r = 0.64). The ArcLength_C is positively related to the calcification angle (r = 0.93), and the ArcLength_F is negatively related to the calcification angle (r = −0.80).

**Figure 4.**
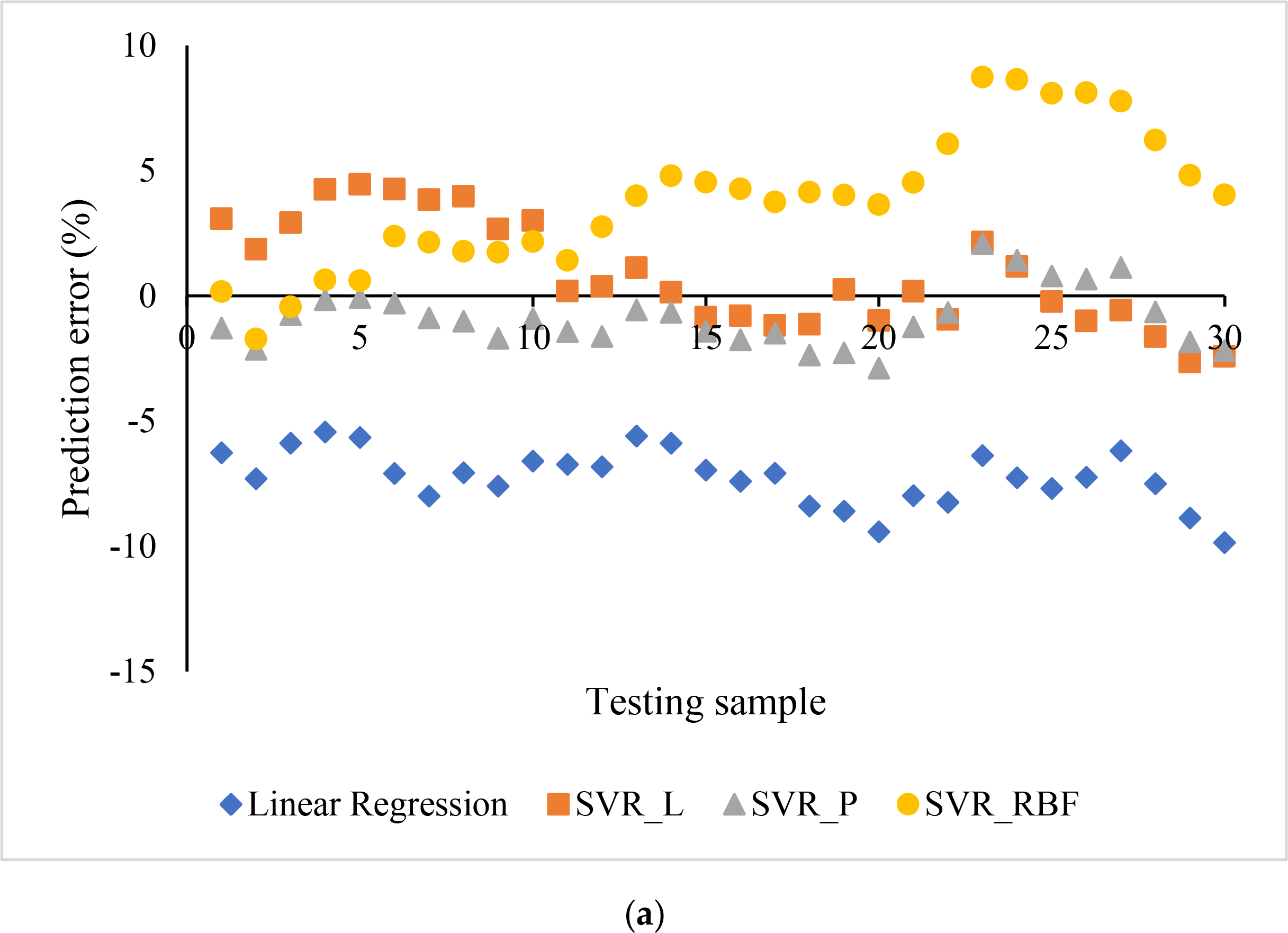

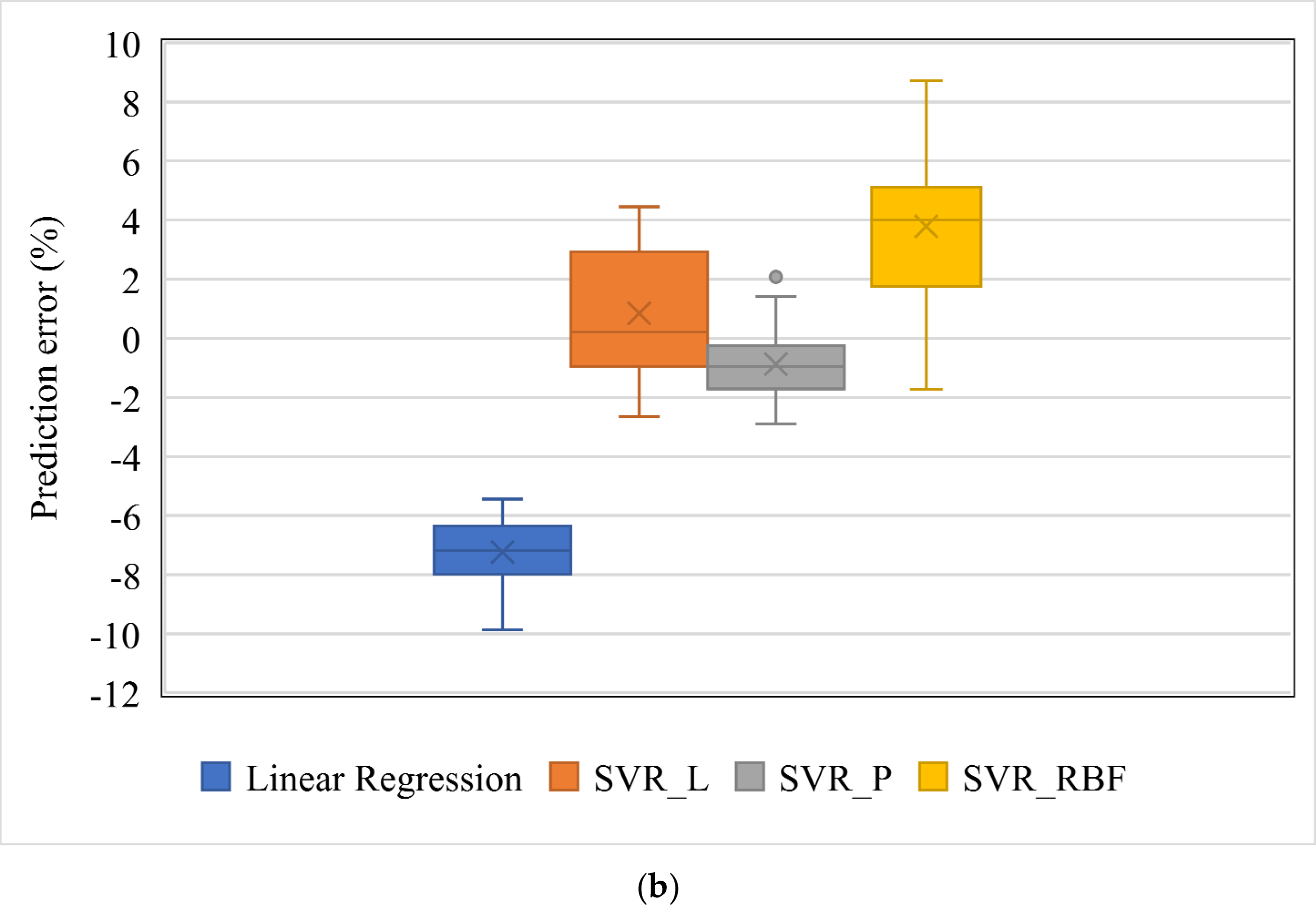
Comparison of linear regression (LR) models and SVR models with linear (SVR_L), polynomial (SVR_P), and radial basic function (SVR_RBF) kernels: (**a**) scatter plot, and (**b**) box plot with median line and mean X.

The prediction errors of four machine learning models are shown in Figure 4 in both scatter and box plots. The errors ranged from −9.85 to −5.44% for the linear regression (LR), −2.46 to 4.45% for SVR with a linear kernel (SVR_L), −2.89 to 2.08% for SVR with a polynomial kernel (SVR_P), and −1.73 to 8.73% for SVR with an RBF kernel (SVR_RBF). The bias values, i.e., the average values of prediction errors, were −7.24, 0.8446, −0.87, and 3.79% for the linear regression and the SVR with linear kernel, polynomial kernel, and RBF kernel, respectively. The ANOVA test showed significantly different prediction errors among these four groups (F = 176.06, *p* < 0.01).

The maximum differences between the ML prediction and the FE simulation, in terms of area, were 0.32, 0.25, 0.22, and 0.16 mm^2^ for the linear regression, the SVR with linear kernel, polynomial kernel, and RBF kernel, respectively. They were much lower than the area difference between the target lumen (7.06 mm^2^) and under expanded one (<5.65 mm^2^).

**Figure 5.**
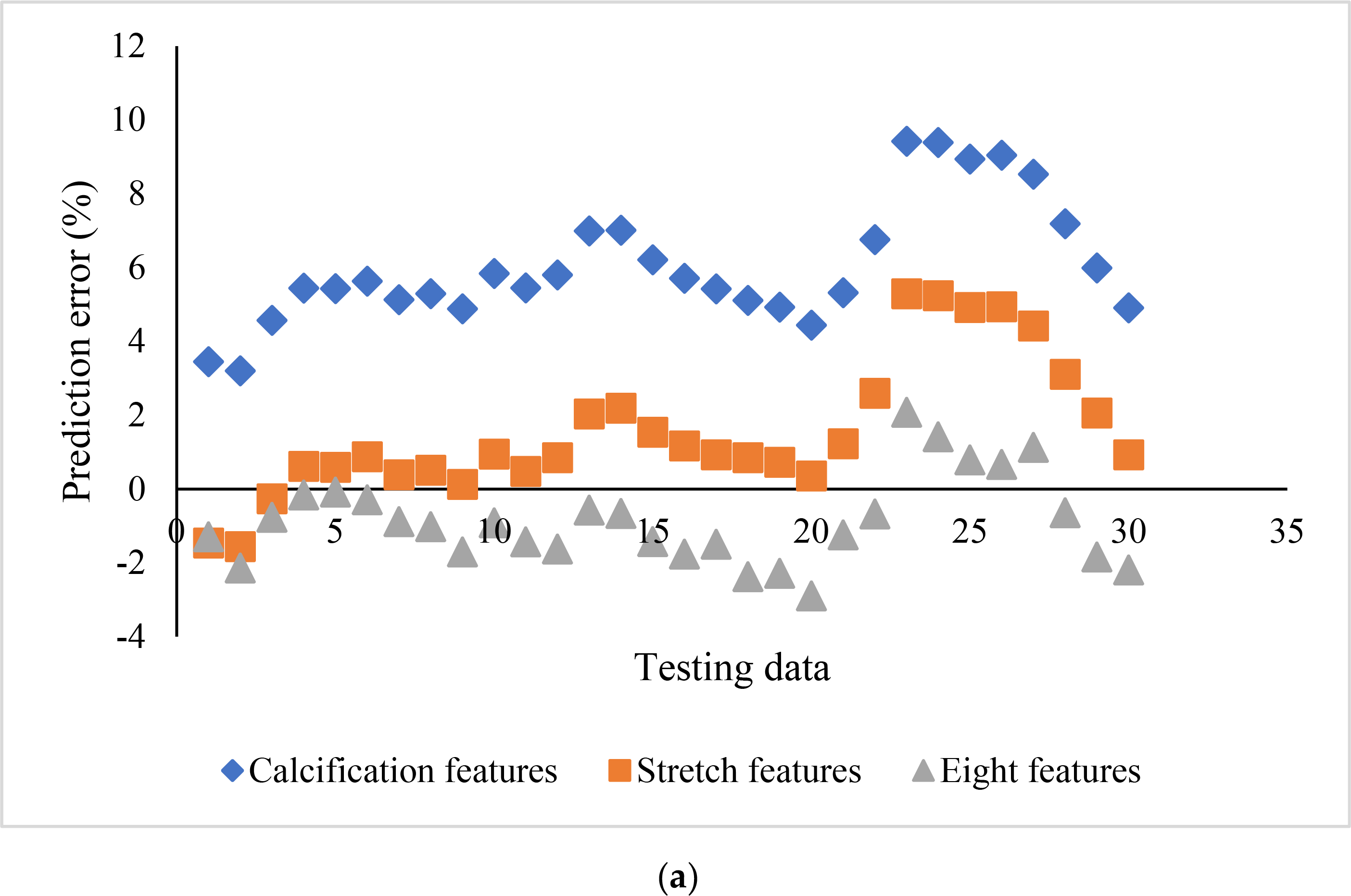

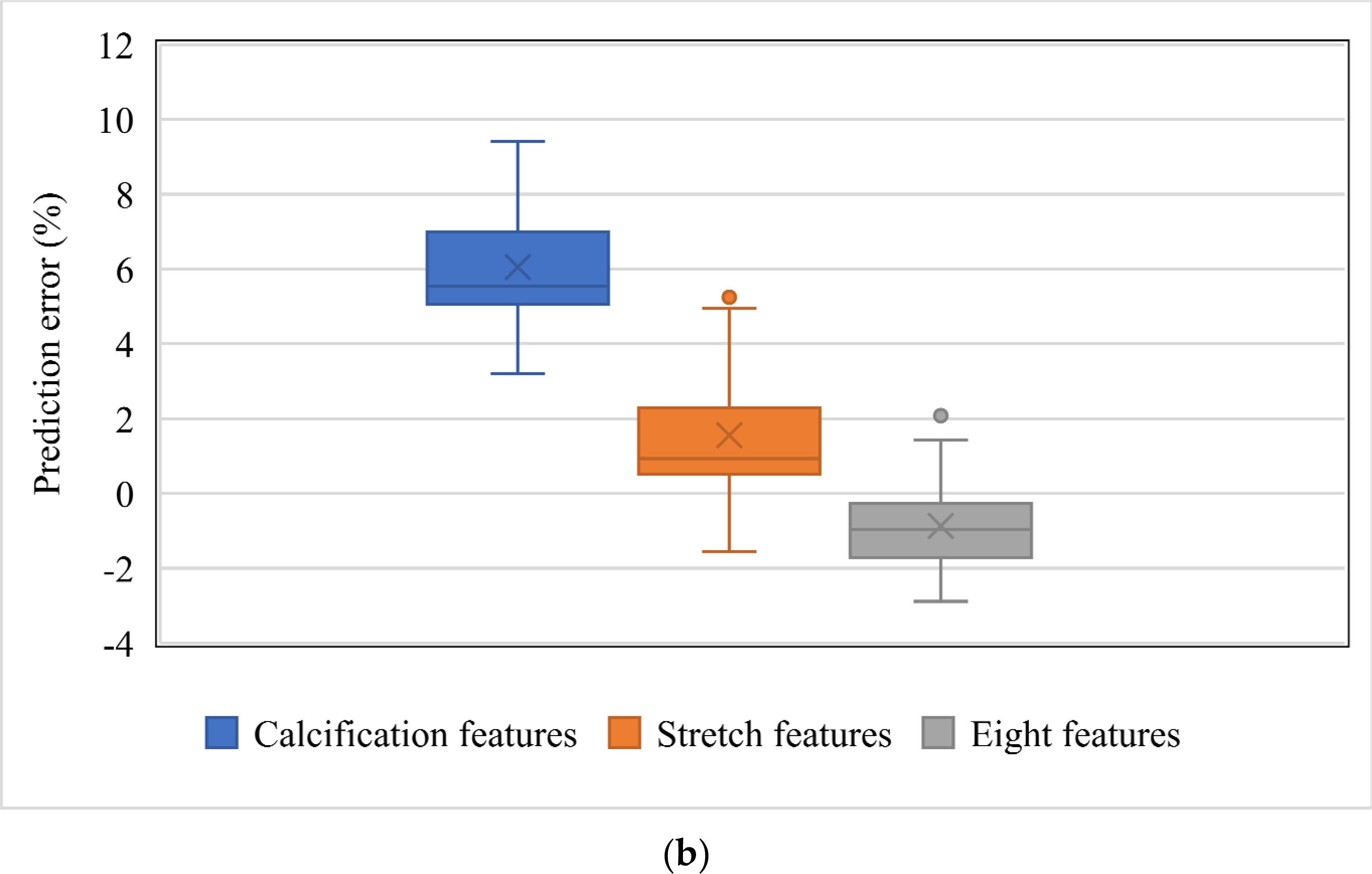
Influence of feature selection on SVR-P prediction errors: (**a**) scatter plot, and (**b**) box plot.

The influence of the feature selection on the prediction accuracy using SVR_P is depicted in Figure 5. Two subgroups, stretch features (Area_L, ArcLength_C, and ArcLength_F) and calcification features (Area_C, Angle_C, and Thickness_C), were compared with the group with all eight features. For the subgroup with calcification features, the prediction error ranged from 3.20 to 9.42%, with the bias as 6.05%. For the subgroup with stretch features, the error ranged from −1.56 to 6.18%, with the bias as 1.56%. For the group with all eight features, the error ranged from −2.89% to 2.08%, with the bias as −0.87%. The group with all eight features demonstrated a better prediction capability than both subgroups. It is interesting to see that the stretch features performed better than the calcification features. The difference among the biases of these three groups is statistically significant (F = 148.9, *p* <0.01). Further, a simple t-test also demonstrated a statistically significant difference between stretch features and calcification features (t = 66.97, *p* < 0.01).

**Figure 6.**
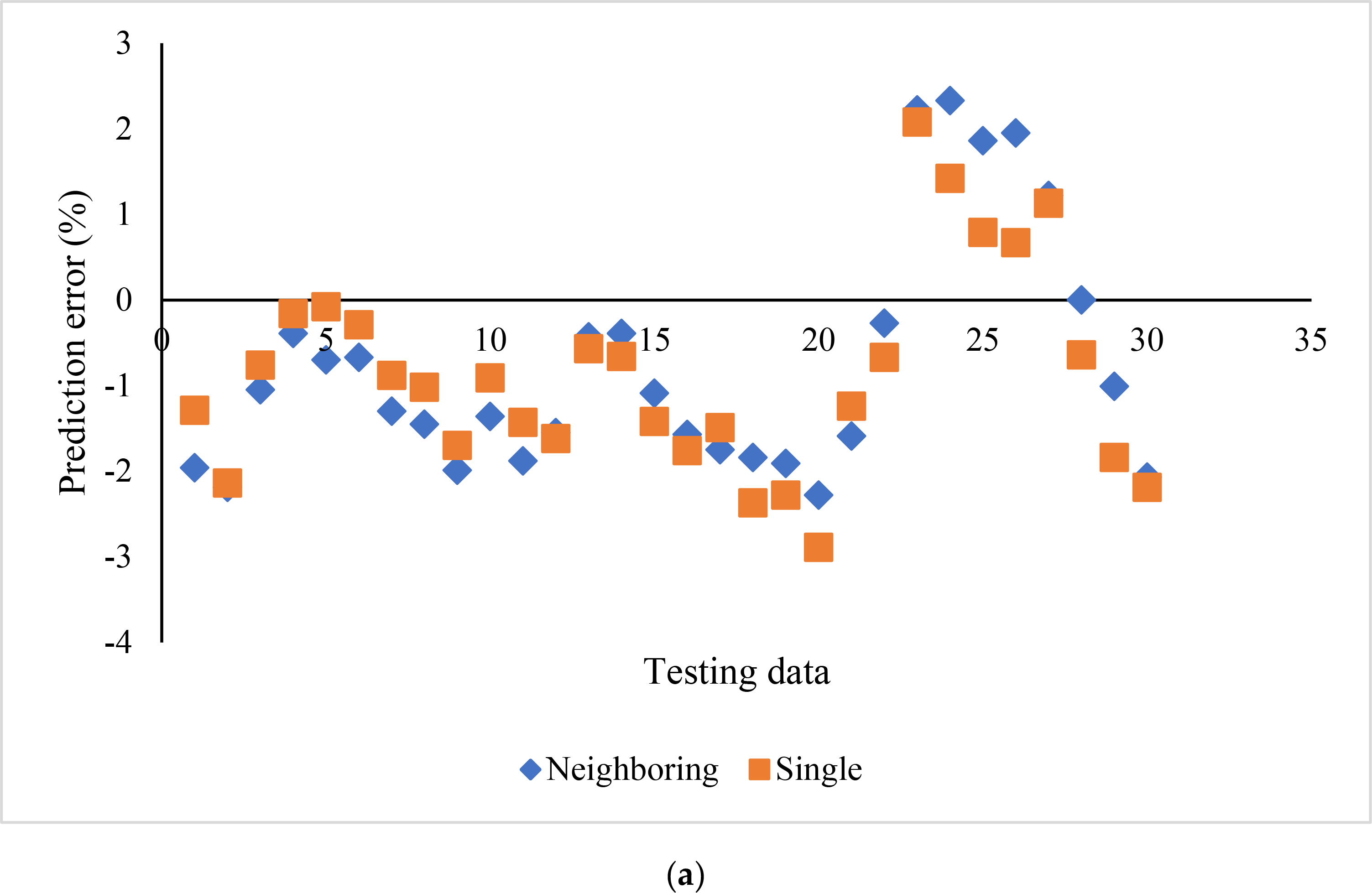

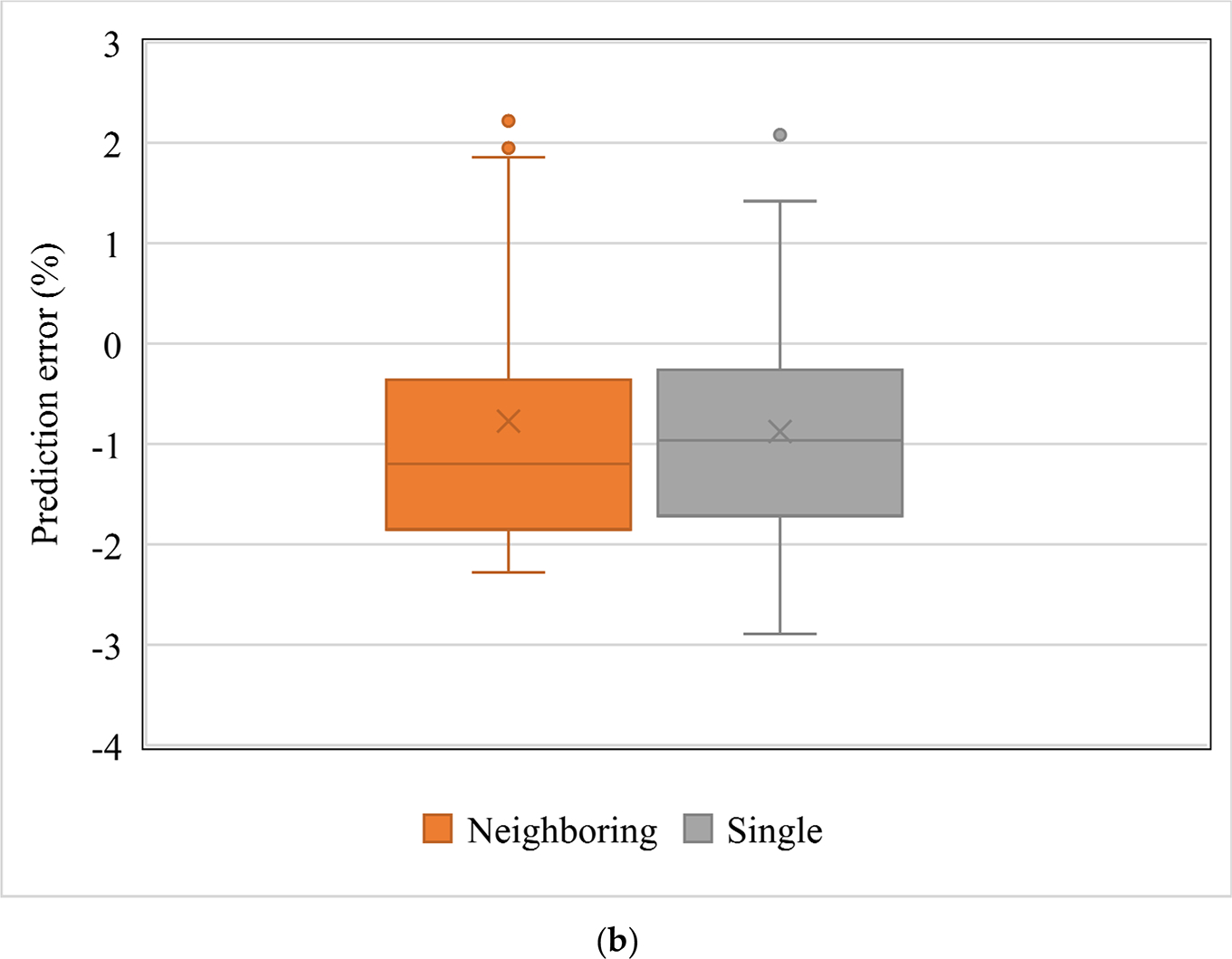
Influence of neighboring cross sections on SVR_P prediction errors: (**a**) scatter plot, and (**b**) box plot.

The influence of neighboring cross sections on the prediction accuracy using SVR_P was evaluated and compared with the aforementioned individual cross sections (Figure 6a). Each feature in the cross section was averaged with the values from eight neighboring cross sections. The prediction errors for both cases showed minimal differences in terms of range and average. Specifically, the prediction error ranged from −2.28 to 2.33% by using averaged features. It ranged from −2.89 to 2.08% by using features from individual cross sections. The bias was −0.77% and −0.87% for neighboring cross sections and single cross sections, respectively. The t-test also showed that there was no significant difference between the biases of these two sets of prediction errors (t = 1.10, *p* = 0.14).

## 4. Discussion

In this work, we developed simulation-driven ML models to predict stent expansion in a calcified coronary artery. The validated FE models saved efforts for post-stenting labeling and provided better feature selection based on mechanistic understanding. Specifically, the FE simulation guided us to select stretch features (Area_L, ArcLength_C, and ArcLength_F) besides classical calcification features (Area_C, Angle_C, and Thickness_C) [2]. Eight pre-stenting geometric features and the post-stenting lumen area were used for training and testing the ML models. The predicted lumen areas from ML models were compared with the ones from FE models. Results have shown that the SVR models with nonlinear kernels provided better predictions. The prediction accuracy of ML in this work was attributed to both our feature selection and the training dataset.

Existing studies have focused on the relationships between calcification attributes and stenting outcomes without considering other relevant features of the artery. Specifically, the retrospective studies provided either associations between calcification attributes and stent expansion [1], or the probability of stent underexpansion using a calcium scoring system [8]. Our recent OCT-based FE models inspected the underlying mechanism of lumen gain. We identified the stretch of fibrotic tissue as the major index of lumen gain following stenting [2]. These lumen features, specifically ArcLength_C and ArcLength_F, were then adopted and tested in our machine learning models via subgroups: calcification feature group and stretch group. The stretch features alone performed better than the calcification features. This indicates that the mechanistic understandings from FE models enable us to identify better features for enhancing the ML analysis. In addition, the validated FE models saved efforts for post-stenting labeling and provided feature selection based on mechanistic understanding.

The prediction accuracy depends on how good training datasets could capture the major features of testing cases. We used one segment of the artery for training (first 90 images out of 120), and the remaining segment for testing. Even though the major characteristics of these images were different (Figure 2), the independence of the testing dataset within one vessel might be limited. Thus, we experimented with various training choices considering the limited dataset. We randomly chose 90 images from this artery, and then the remaining 30 images of the same artery were used for testing. The prediction accuracy slightly increased, compared with the aforementioned segment training and testing (supplemental Figure S1). Moreover, our simulation-driven ML framework was further examined in three more patient-specific arterial models with different calcification attributes. First, we mixed all cross section images from three vessels, and used 75% of randomly drawn images for training. Then, we used cross section images from three vessels for training, and the images from the fourth vessel for testing. The prediction accuracy of both experiments declined further (data not shown for brevity). This was expected since the training dataset was not large enough to capture the major features of the testing case.

We also examined neighboring cross sections to avoid potential imaging artifacts. No statistically significant differences were observed in terms of the bias and range of the prediction errors. This indicated that individual cross sections are capable of predicting the post-stenting lumen area.

There are limitations to our work. In machine learning, more data is almost always advised. In our case, we trained and tested on data from different segments of one vessel. This might not adequately test generalizability. Nevertheless, this report does show the feasibility of integrating machine learning and finite element models to identify appropriate features for predicting stent expansion. Future work will include more training/testing data, allowing us to assess generalizability. The framework in this work could be expanded to incorporate more features including pre-stenting strategies and post-dilation parameters, such as balloon diameter and inflation pressure, which will guide clinicians in determining a better surgical plan. Using 3D features might help capture the full range of calcification lesion responses, especially when there are highly irregular lesions. The lesion stress or strain could also be used as prediction outcomes to estimate the risk of calcification fracture and tissue dissection.

## 5. Conclusions

In this work, we developed simulation-driven machine learning models to predict stent expansion in a calcified coronary artery. The mechanistic depiction of the stent–artery interaction guided us to identify better features for predicting the post-stenting lumen area. Results showed that geometric features from a single cross section image are capable of predicting the post-stenting lumen area. In addition, the SVR models with nonlinear kernels provided better predictions. Neighboring cross sections, compared with individual cross sections, could improve the prediction stability. The simulation-driven ML framework developed in this work could be extended to a large amount of vessel datasets, which may pave the way toward better prediction of stenting outcomes in specific lesions.

## Supplementary Material

Figure S1

## Figures and Tables

**Table 1.** Material coefficients.

Tissue	C_10_ (MPa)	C_01_ (MPa)	C_11_ (MPa)	C_20_ (MPa)	C_02_ (MPa)	C_30_ (MPa)	C_03_ (MPa)

Artery	0.10881	−0.101	−0.1790674	0.0885618	0.062686		
Fibrotic tissue	0.04				0.003		0.02976
Calcification	−0.49596	0.50661	1.19353	3.6378		4.73725	
