# Supplementary material for: Simulation-Driven Machine Learning for Predicting Stent Expansion in Calcified Coronary Artery
Source: Appl Sci (Basel). Author manuscript; Available in PMC 2022 Jul 27. (PMC9328568; doi:10.3390/app10175820)
Supplement: Figure S1 [file NIHMS1822446-supplement-Figure_S1.pdf]

applied sciences

Supplementary Materials: Simulation-Driven Machine Learning for Predicting Stent Expansion in Calcified Coronary Artery

Pengfei Dong, Guochang Ye, Mehmet Kaya and Linxia Gu

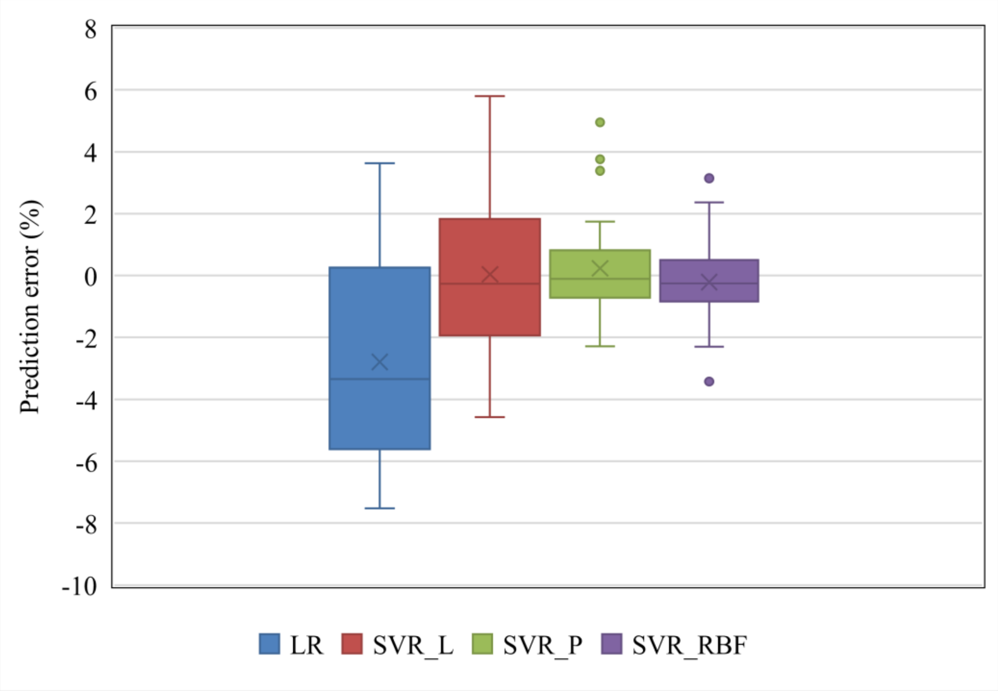

Figure S1. Prediction errors of ML methods with randomly drawn training dataset. The errors ranged from −7.51 to 3.63% for the linear regression (LR), −4.57 to 5.80% for SVR with a linear kernel (SVR_L), −2.29 to 4.95% for SVR with a polynomial kernel (SVR_P), and −3.43 to 3.25% for SVR with an RBF kernel (SVR_RBF). The biases were −2.79, 0.04, 0.23, and −0.21% for the linear regression and the SVR with linear kernel, polynomial kernel, and RBF kernel, respectively. The ANOVA test showed significantly different prediction errors among these four groups ($F = 11.09$, $p < 0.01$).
